# *Hibiscus syriacus* Bud ‘Pyeonghwa’ Water Extract Inhibits Adipocyte Differentiation and Mitigates High-Fat-Diet-Induced Obesity In Vivo

**DOI:** 10.3390/ijms26209870

**Published:** 2025-10-10

**Authors:** Shin-Hye Kim, Hye-Lim Shin, Tae Hyun Son, Dongsoo Kim, Hae-Yun Kwon, Hanna Shin, Yunmi Park, Sik-Won Choi

**Affiliations:** 1 Forest Biomaterials Research Center, National Institute of Forest Science (NIFoS), Jinju 52817, Republic of Korea; black7a@korea.kr (S.-H.K.); hlims0901@korea.kr (H.-L.S.); snoopyegg@korea.kr (T.H.S.); skimds@korea.kr (D.K.); 2 Department of Forest Bio-Resources, National Institute of Forest Science (NIFoS), Suwon 16631, Republic of Korea; kwonhy05@korea.kr (H.-Y.K.); hanna193@korea.kr (H.S.); 3 Research Planning and Coordination Division, National Institute of Forest Science (NIFoS), Seoul 02455, Republic of Korea; pym5250@korea.kr

**Keywords:** obesity, *Hibiscus syriacus*, adipogenesis, PPARγ, HFD-induced obesity

## Abstract

Obesity, characterized by the accumulation of excess adipocytes, is a significant risk factor for type 2 diabetes and non-alcoholic fatty liver disease. Medicinal plants, including *Hibiscus sabdariffa*, have been traditionally employed to prevent or treat conditions such as obesity and inflammation due to their safety profile and minimal side effects during long-term use. However, the anti-obesity potential of *Hibiscus syriacus*, a taxonomically distinct species within the same genus, remains unexplored. In this study, we screened 181 varieties of *H. syriacus* buds for anti-obesity effects and identified the water extract of the ‘Pyeonghwa’ bud (HPWE) as a potent inhibitor of adipogenesis. Using 3T3-L1 murine pre-adipocyte cells, we demonstrated that HPWE significantly reduced lipid accumulation without inducing cytotoxicity. Mechanistically, HPWE downregulated the expression of key adipogenic signaling proteins and transcription factors, including peroxisome proliferator-activated receptor gamma (PPARγ) and CCAAT/enhancer-binding protein alpha (C/EBPα), which serve as molecular markers of adipogenesis. Additionally, in vivo experiments employing a high-fat-diet-induced obesity mouse model using C57BL/6 species confirmed the anti-obesity effects of HPWE. Collectively, these findings suggest that HPWE represents a promising candidate for the prevention of obesity.

## 1. Introduction

The World Health Organization reported that, in 2016, 1.9 billion people with overweight were aged 18 years and older, of which 650 million were classified as obese. The mortality rate among overweight and obese populations is higher than that among underweight populations. According to the 2025 edition of the UNICEF/WHO/World Bank Group Joint Child Malnutrition Estimates (JME), 35.5 million children under five years of age were classified as overweight globally in 2024 [1]. The body mass index (BMI), calculated by dividing an individual’s body weight by the square of their height, is widely acknowledged as the primary diagnostic measure for assessing obesity [2]. According to the World Health Organization (WHO), overweight and obesity are defined as BMIs ranging from 25 to 30 kg/m^2^ and greater than 30 kg/m^2^, respectively [3]. Excessive fat accumulation results in weight gain and obesity, which significantly elevate the risk of adverse health outcomes. Consequently, obesity represents one of the most significant global threats to morbidity and mortality [4,5]. There is an urgent need for novel therapeutic interventions to address the unmet medical challenges associated with reversing this trend [6]. Obesity is associated with metabolic disorders such as type 2 diabetes and non-alcoholic fatty liver disease and is a risk factor for coronary artery disease, stroke, chronic obstructive pulmonary disease, lung cancer, chronic liver disease, and both acute and chronic renal failure [7]. The excessive energy metabolism resulting from adipose tissue accumulation contributes to weight gain and obesity. Adipose tissue functions primarily as an active endocrine organ that secretes adipokines and stores energy [8]. Overweight and obesity typically arise from unhealthy lifestyle behaviors, including excessive consumption of high-calorie foods, frequent eating, and snacking after meals [9]. An increasing number of individuals with obesity are seeking pharmacological treatments such as liraglutide, which reduces hunger, and orlistat, which inhibits fat absorption. However, some of these medications are associated with significant side effects and long-term safety concerns, including nausea, vomiting, satiety, and oily feces [10].

Excess energy is converted into lipids through the process of adipogenesis, with these lipids subsequently being stored within adipocytes [11]. During adipogenesis, mesenchymal stem cells (MSCs) differentiate into adipocytes [12]. This differentiation is regulated by complex signaling pathways involving adipogenesis-specific transcription factors and proteins [13,14,15,16], which also serve as molecular biomarkers of adipogenesis [11,17]. An excess of differentiated adipocytes leads to the accumulation of dispersed fat, resulting in an increase in adipocyte number (hyperplasia) and/or size (hypertrophy), thereby contributing to obesity [18]. The 3T3-L1 mouse preadipocyte cell line is extensively utilized to investigate the molecular mechanisms underlying preadipocyte development [19]. Additionally, mice fed a high-fat diet (HFD) are commonly employed as an experimental model of obesity, as such diets closely mimic the etiological factors of human obesity [20]. Early stages of HFD-induced obesity in mice are characterized by expansion of epididymal white adipose tissue (WAT) [21]. Lipogenesis, encompassing fatty acid and triglyceride synthesis, also plays a significant role in WAT growth [22]. The HFD model demonstrates adipose tissue expansion through both hyperplasia and hypertrophy [23]. Consequently, strategies aimed at inhibiting adipocyte differentiation and mitigating HFD-induced obesity are critical for the identification of effective anti-obesity agents.

Medicinal herbs are often considered alternatives to conventional therapies for the management and prevention of various diseases because of their favorable safety profile for long-term use and minimal side effects [24]. *Hibiscus syriacus* L. (Rose of Sharon), the national flower of Korea, also carries significant cultural importance in the region [25]. Flower buds of *H. syriacus* have been reported to provide various health benefits, including antihypertensive and anticancer effects [26,27]. In addition, previous research has shown that *Hibiscus sabdariffa* (Roselle), commonly consumed as tea, exhibits antioxidant and anti-obesity properties [28,29]. However, the anti-obesity potential of *H. syriacus*, a species closely related to *H. sabdariffa*, has not yet been investigated. Consequently, this study evaluated the anti-obesity effects of flower buds from 181 varieties of *H. syriacus*. The buds of the selected ‘Pyeonghwa’ variety were further analyzed to elucidate their molecular mechanisms and mode of action using an in vivo high-fat-diet-induced obesity model.

## 2. Results

### 2.1. HPWE Suppresses Lipid Accumulation in 3T3-L1 Cells

**Figure 1:**
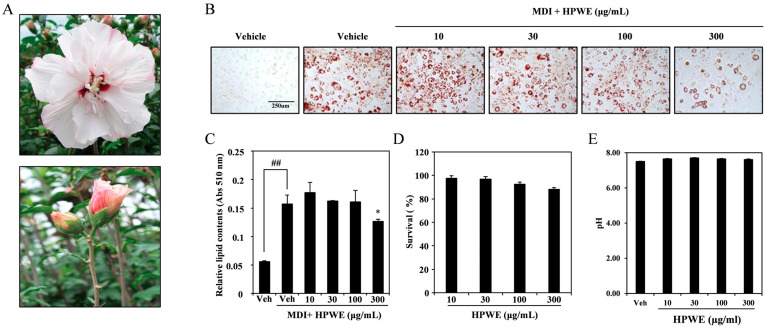
The water extract of *Hibiscus syriacus* bud ‘Pyeonghwa’ (HPWE) reduces lipid accumulation in 3T3-L1 cells. (**A**) Image of *Hibiscus syriacus* ‘Pyeonghwa’ buds. (**B**) Representative images depicting HPWE-induced differentiation of 3T3-L1 pre-adipocytes. scale bar is 250 μm. (**C**) Quantitative analysis corresponding to panel (**B**). Statistical significance is indicated as ## *p* < 0.01 (compared to negative control) and * *p* < 0.05 (compared to positive control). Data are presented as mean ± SD (n = 3). Comparisons between two groups (negative control vs. positive control, or positive control vs. treatment) were analyzed using Student’s *t*-test. (**D**) Viability of 3T3-L1 cells following HPWE treatment, measured by absorbance (Abs). (**E**) Changes in the pH of culture media expressed as a percentage following HPWE treatment.

To evaluate the anti-adipogenic effects of the water extract derived from *H. syriacus* ‘Pyeonghwa’ buds (HPWE; Figure 1A), 3T3-L1 cells were induced to differentiate into adipocytes using medium supplemented with MDI. HPWE inhibited adipogenesis in a dose-dependent manner (Figure 1B). Quantitative analysis of lipid content demonstrated that while MDI promoted adipocyte differentiation, HPWE significantly reduced lipid accumulation (Figure 1C). Furthermore, the concentrations of HPWE employed in this study did not exhibit cytotoxic effects (Figure 1D), and the pH of the culture medium remained stable following HPWE treatment (Figure 1E). Collectively, these findings indicate that HPWE exerts anti-adipogenic effects on 3T3-L1 cells without inducing cytotoxicity and disrupting the pH stability.

### 2.2. HPWE Attenuates the Expression of Molecular Markers Associated with Adipocyte Differentiation in 3T3-L1 Cells

**Figure 2:**
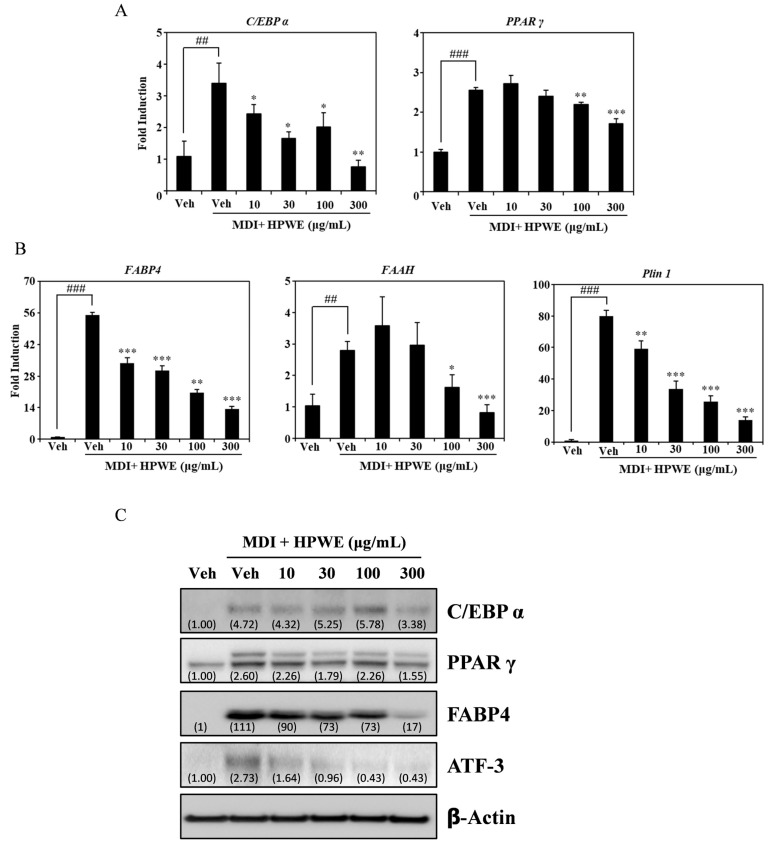
*Hibiscus syriacus* bud ‘Pyeonghwa’ water extract (HPWE) inhibits the expression of adipogenic markers in 3T3-L1 cells. (**A**) RT-qPCR analysis of mRNA expression levels of transcription factors involved in adipocyte differentiation in 3T3-L1 cells treated with vehicle or varying concentrations of HPWE (10, 30, 100, and 300 μg/mL). *β-Actin* served as the internal control. (**B**) RT-qPCR analysis of mRNA expression of molecular markers associated with adipocyte differentiation under the same treatment conditions. *β-Actin* was used as the internal control. Statistical significance is indicated as follows: ## *p* < 0.01 and ### *p* < 0.001 compared to the negative control; * *p* < 0.05, ** *p* < 0.01, and *** *p* < 0.001 compared to the positive control. Data are presented as mean ± SD (n = 3). Comparisons between two groups (negative control vs. positive control, or positive control vs. treatment) were analyzed using Student’s *t*-test. (**C**) Western blot analysis demonstrating the effects of HPWE on protein levels of adipogenesis-related markers, with actin as the loading control. A representative result from three independent experiments with consistent findings is shown.

To elucidate the pathway through which HPWE regulates adipogenesis, we employed RT-qPCR to assess the expression of molecular markers involved in adipocyte differentiation. HPWE treatment resulted in a dose-dependent inhibition of mRNA expression of key transcription factors associated with adipogenesis, including *PPARγ* and *C/EBPα* (Figure 2A). Additionally, HPWE suppressed the mRNA levels of adipogenesis-related markers such as *FABP4*, *FAAH*, and *PLin1* in a dose-dependent manner (Figure 2B). Western blot analysis further demonstrated that HPWE treatment reduced the expression of proteins involved in adipocyte differentiation (Figure 2C). Collectively, these findings indicate that HPWE inhibits adipogenesis by downregulating the expression of adipocyte differentiation markers.

### 2.3. HPWE Inhibits Body Weight Gain in Mice Fed a High-Fat Diet

**Figure 3:**
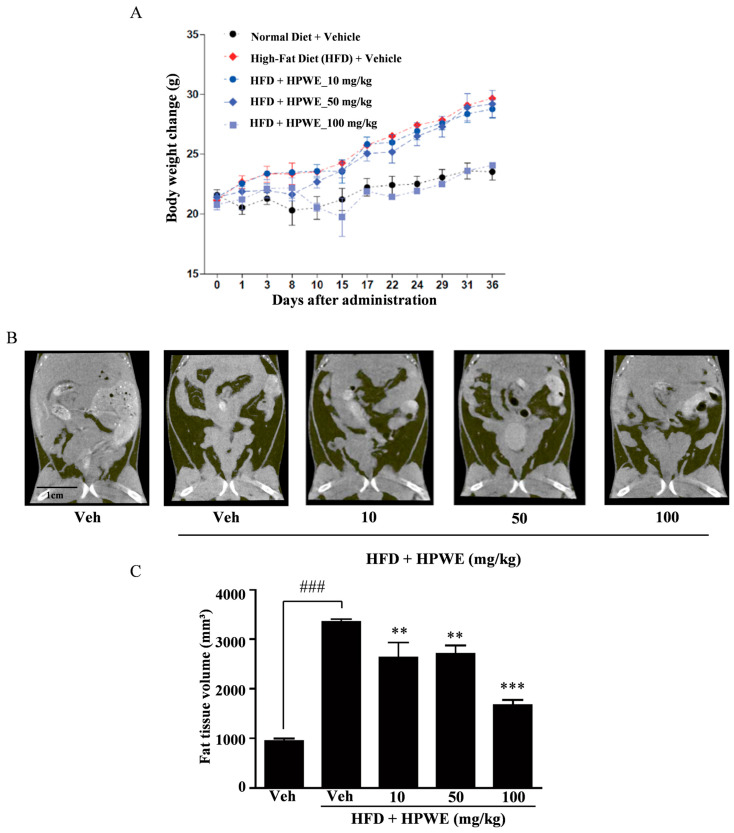
*Hibiscus syriacus* bud ‘Pyeonghwa’ water extract (HPWE) inhibits body weight gain in mice fed a high-fat diet (HFD). (**A**) Body weight changes over the 36-day experimental period were assessed in mice fed a normal diet with vehicle (black circles), a high-fat diet (HFD) with vehicle (red diamonds), or an HFD supplemented with HPWE at doses of 10 mg/kg (blue circles), 50 mg/kg (blue diamonds), or 100 mg/kg (light blue squares). Data are presented as mean ± standard deviation (n = 5). (**B**) Representative micro-CT images depicting whole-body fat in mice following HPWE treatment. scale bar is 1 cm. (**C**) Quantitative analysis of the data shown in panel (**B**). Data are presented as mean ± SD (n = 5). Comparisons among multiple groups (negative control, HFD-fed control, and HPWE concentrations) were analyzed using one-way ANOVA followed by Tukey’s post hoc test. Statistical significance is indicated as follows: ### *p* < 0.001 compared to the negative control; ** *p* < 0.01 and *** *p* < 0.001 compared to the positive control. Veh denotes vehicle control.

We conducted experiments using mice fed a high-fat diet (HFD) to evaluate the anti-obesity effects of HPWE in vivo. The HPWE-treated group demonstrated a lower rate of weight gain compared to the HFD group (Figure 3A). To further elucidate the anti-obesity effects of HPWE in HFD-fed mice, whole-body fat accumulation was assessed using micro-computed tomography (micro-CT). The HFD group exhibited greater body fat accumulation than the vehicle group (Figure 3B). In contrast, mice treated with HPWE showed a reduction in body fat relative to the HFD group (Figure 3C). These findings indicate that HPWE exerts anti-adipogenic effects in a mouse model of HFD-induced obesity.

### 2.4. HPWE Decreases Epididymal White Adipose Tissue in Subjects Fed a High-Fat Diet

**Figure 4:**
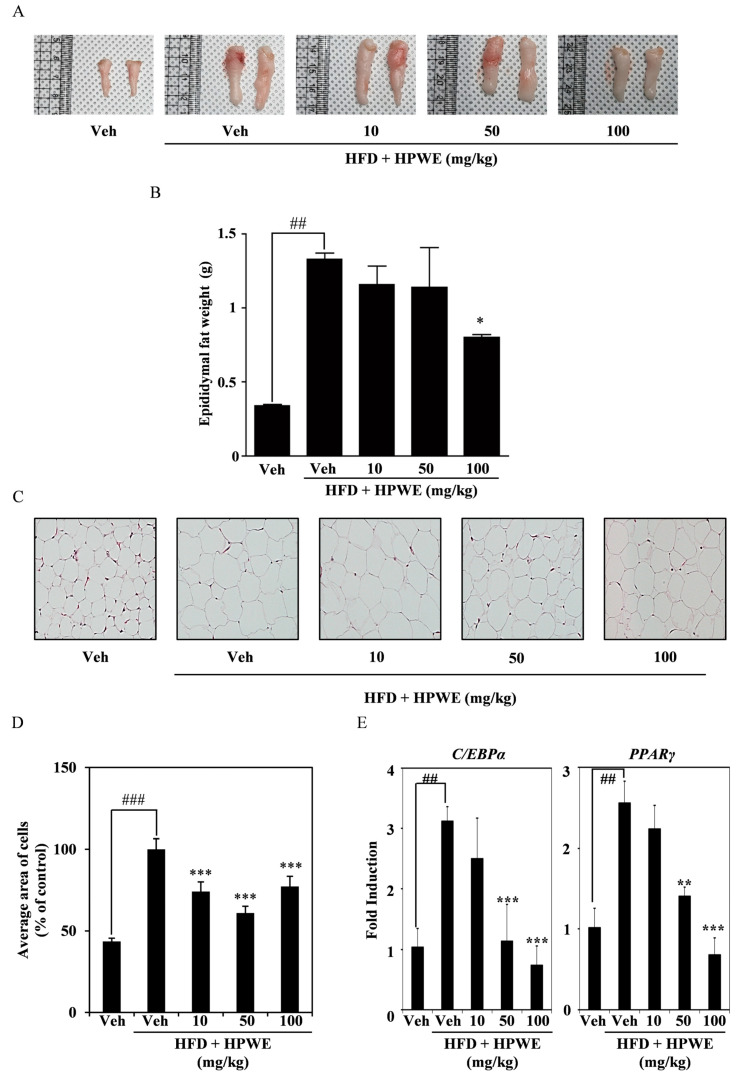
*Hibiscus syriacus* bud ‘Pyeonghwa’ water extract (HPWE) modulates epididymal white adipose tissue accumulation in mice subjected to a high-fat diet. (**A**) Representative images of epididymal white fat tissue following HPWE administration. (**B**) Quantitative analysis of epididymal white fat tissue mass (n = 5). Data are presented as mean ± SD (n = 5). Comparisons among multiple groups (negative control, HFD-fed control, and HPWE concentrations) were analyzed using one-way ANOVA followed by Tukey’s post hoc test. Statistical significance is indicated as follows: ## *p* < 0.01 compared to the control; * *p* < 0.05 compared to the HFD-fed control. (**C**) Representative hematoxylin and eosin-stained sections of epididymal white fat tissue from HPWE-treated mice. Magnification: 200×. (**D**) Mean adipocyte size in epididymal white fat tissue of HPWE-treated mice. Data are presented as mean ± SD (n = 5). Comparisons among multiple groups (negative control, HFD-fed control, and HPWE concentrations) were analyzed using one-way ANOVA followed by Tukey’s post hoc test. Statistical significance is indicated as follows: ### *p* < 0.001 compared to the control; *** *p* < 0.001 compared to the HFD-fed control. (**E**) After experiment period, the mRNA expression of transcription factors involved in adipocyte differentiation was assessed using qRT-PCR in epididymal WAT stimulated with either vehicle or HPWE at 10, 50, 100 mg/kg. β-actin served as the internal control. Data are presented as mean ± SD (n = 5). Two-group comparisons were analyzed using Student’s *t*-test. ## *p* < 0.01 (versus control), ** *p* < 0.01, *** *p* < 0.001 (versus HFD-fed control).

To provide more detailed evidence of the effect of HPWE on reducing body weight, epididymal white adipose tissue (WAT) was harvested and analyzed. As shown in Figure 4, HPWE treatment significantly decreased both the volume (Figure 4A) and weight (Figure 4B) of epididymal WAT. To evaluate adipocyte size, epididymal fat samples were collected and subjected to hematoxylin and eosin staining. The hypertrophied adipocytes observed in the high-fat diet group were markedly reduced following HPWE administration (Figure 4C,D). To elucidate the molecular mechanisms underlying epididymal white adipose tissue, RNA was extracted from this tissue, and quantitative reverse transcription polymerase chain reaction (qRT-PCR) was conducted. The results indicated that HPWE inhibited PPAR gamma and C/EBP alpha in a dose-dependent manner (Figure 4E). These results suggest that HPWE exerts anti-obesity effects in a mouse model of HFD-induced obesity.

### 2.5. LC-MS Profiling of Saponarin in HPWE

**Figure 5:**
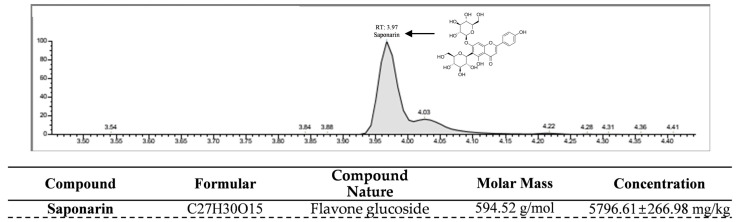
Quantitative Analysis of Saponarin Content in HPWE using LC-MS/MS. A saponarin ion chromatogram of HPWE produced by Liquid chromatography-mass spectrometry (LC-MS/MS) analysis.

To evaluate the presence of saponarin, a potential anti-adipogenic compound, in HPWE, we conducted LC-MS analysis to profile its constituents. As shown in Figure 5, a peak with a retention time of 3.97 min, tentatively identified as saponarin, was observed in the negative ion-mode LC-MS chromatogram. To quantify the saponarin concentration in the HPWE sample utilized in this study, a standard calibration curve was established (Supplementary Figure S1). The analysis revealed that HPWE contains saponarin at a concentration of 5796.61 mg/kg. Collectively, these findings indicate that HPWE is characterized by a high saponarin content.

## 3. Discussion

In the present study, we demonstrated that treatment with HPWE exerts anti-obesity effects both in vitro and in vivo, without inducing cytotoxicity. To the best of our knowledge, this is the first study to report the anti-obesity properties of HPWE. The significance of our findings lies in the fact that the hot water extract exhibited anti-obesity effects, and since it can be consumed as tea, it offers greater accessibility to the general population.

The investigation of 3T3-L1 cell differentiation serves as an effective model for screening potential anti-obesity agents [19]. In the present study, we evaluated the anti-obesity effects of extracts derived from 181 varieties of *H. syriacus* buds. Among these, the extract from the ‘Pyeonghwa’ variety exhibited the most potent anti-obesity activity in 3T3-L1 cells. Specifically, HPWE inhibited lipid accumulation in 3T3-L1 cells, which are murine pre-adipocytes known to accumulate lipids during differentiation. Previous research has explored the anti-obesity properties of various Hibiscus species [30]. Notably, these studies have identified numerous flavonoids, anthocyanins, organic acids, and phenolic acids with demonstrated anti-obesity effects in *Hibiscus* spp. [28]. In this study, flower buds were selected as the material of interest due to their traditional use in diverse medicinal applications [31].

Adipocyte differentiation necessitates the expression of multiple transcription factors, notably PPARγ and C/EBPα [32,33]. The process of adipogenesis is initiated through the interaction between C/EBPα and PPARγ [34]. Consequently, the inhibition of PPARγ and C/EBPα represents a potential strategy for the prevention and treatment of obesity. In this study, we observed that HPWE suppresses the expression of PPARγ and C/EBPα at both the transcriptional and protein levels. Consistent with these findings, previous research on the anti-obesity effects of *Hibiscus sabdariffa* extract demonstrated that its inhibitory action is mediated via PPARγ and C/EBPα [35]. Additional studies have identified several other markers associated with adipogenesis. Specifically, the inhibition of PPARγ and C/EBPα in 3T3-L1 cells leads to a downregulation of their differentiation [36]. While PPARγ and C/EBPα are implicated in the early stages of 3T3-L1 cell differentiation, FABP4 and PLIN1 are involved in the intermediate phase [37]. FABP4 is markedly expressed throughout adipocyte differentiation and is transcriptionally regulated by PPARγ [38]. The mammalian integral membrane enzyme FAAH is responsible for the degradation of fatty acid amide endogenous signaling lipids [39]. Notably, FAAH expression is upregulated by a high-fat diet, whereas treatment with germinated soy germ extract, which exhibits anti-obesity properties, reduces FAAH expression [40]. In the present investigation, HPWE was found to inhibit the expression of both FABP4 and FAAH. Furthermore, PLIN1 facilitates the expression of genes related to lipid metabolism at the mRNA and protein levels [14]. High-density lipoprotein induces activation of the transcription factor ATF-3 as a target gene in macrophages [41]. Our results indicate that HPWE treatment attenuates the expression of PLIN1 and ATF-3, thereby supporting the hypothesis that HPWE exerts anti-obesity effects in vitro. Collectively, these findings suggest that HPWE reduces adipocyte lipid accumulation through the inhibition of PPARγ and C/EBPα, as well as by suppressing multiple markers involved in adipocyte differentiation.

Numerous mouse models of obesity have been developed previously. The first is an HFD-fed mouse model, and the second is a leptin-deficient (ob/ob) mouse model. Although the latter does not accurately represent human obesity, diet-induced obesity models are considered more representative of the human condition [42]. Therefore, we selected the HFD mouse model for our study. HFD feeding resulted in body weight gain, which was inhibited by treatment with HPWE. Notably, the HPWE-treated group exhibited a reduction in abdominal fat tissue. Epididymal white adipose tissue (WAT) secretes various cytokines that regulate metabolism in organs and tissues during HFD-induced obesity [43]. Our results demonstrated that both the size and weight of epididymal WAT decreased following HPWE treatment. Furthermore, a five-week treatment with HPWE significantly reduced the level of epididymal WAT. The growth of epididymal WAT is observed at an early stage in mice with diet-induced obesity [21]. The reduction in epididymal WAT mass is attributed to decreases in both the size and number of adipocytes [44]. The mRNA expression levels of PPARγ and C/EBPα in epididymal WAT were observed to decrease following treatment with HPWE. Collectively, these findings suggest that HPWE inhibits adipocyte differentiation both in vitro and in vivo in high-fat diet-induced obesity through the suppression of PPARγ.

*H. syriacus* contains several bioactive compounds, including o-coumaric acid, p-coumaric acid, schaftoside, isoschaftoside, apigenin-6-C-glucoside-7-O-glucoside (saponarin), and kaempferol-3-O-galactoside-7-O-rhamnoside. Notably, saponarin is recognized for its antioxidant properties and hepatoprotective effects [25]. To date, research on saponarin derived from *H. syriacus* has primarily concentrated on its potential to ameliorate sleep disorders [45]. However, studies have also demonstrated that saponarin exhibits anti-obesity effects, as evidenced by both in vitro and in vivo investigations. Specifically, analyses of hot water extract of barley sprouts (BSE), which are rich in saponarin, have shown the ability to inhibit the differentiation of 3T3-L1 pre-adipocytes into mature adipocytes [46]. Quantitative analysis of saponarin content in HPWE confirmed a concentration of 5796.61 mg/kg. In conclusion, this study verified that HPWE inhibits lipid accumulation by suppressing adipocyte differentiation and proposed that it represents a functional natural product with potential applications in body weight management.

## 4. Materials and Methods

### 4.1. Preparation of HPWE

Experimental materials were collected and used to prepare *H. syriacus* flower buds. *H. syriacus* bud was collected from the Division of Special Forest Resources, National Institute of Forest Science (NIFoS, Suwon, Gyeonggi-do, Republic of Korea) in June and July 2020. The plants were washed with clean sterile water and air-dried at 50 °C for 3 d to remove moisture. Next, the dried *H. syriacus* flower buds were treated with deionized water in an autoclave at 121 °C for 15 min. The crude extracts were subjected to centrifugation, followed by filtration and freeze-drying to obtain a dry powder. Subsequently, the HPWE was reconstituted using dimethyl sulfoxide at a concentration of 30 mg/mL (DMSO; Sigma Aldrich, St. Louis, MO, USA).

### 4.2. Reagents and Antibodies

Fetal bovine serum (FBS) and antibiotics (penicillin and streptomycin) were purchased from Gibco (Thermo Fisher Scientific, Waltham, MA, USA). TRIzol was purchased from Invitrogen (Carlsbad, CA, USA). DMSO was obtained from Sigma-Aldrich (St. Louis, MO, USA). Anti-actin and anti-horseradish peroxidase (HRP)-conjugated mouse and rabbit antibodies were purchased from Santa Cruz Biotechnology (Santa Cruz, CA, USA). Antibodies against C/EBPα, PPARγ, FABP4, and ATF-3 were purchased from Cell Signalling Technology (Beverly, MA, USA).

### 4.3. Cell Culture

Mouse 3T3-L1 pre-adipocytes obtained from the American Type Culture Collection (ATCC CL-173™, Manassas, VA, USA) were cultured in Dulbecco’s modified Eagle’s medium supplemented with 10% FBS and antibiotics at 37 °C and 5% CO_2_ atmosphere in an incubator (MCO-170AIC-PK; Panasonic, Osaka, Japan). The medium was changed every 2–3 d. To promote differentiation, the pre-adipocytes were cultured in a medium containing 0.5 mM isobutylmethylxanthine (IBMX), 5 μM dexamethasone, 0.5 μg/mL insulin, and 10% FBS (MDI). IBMX, dexamethasone, and insulin were purchased from sigma Aldrich (St. Louis, MO, USA), and the first day was designated day 0 of differentiation. The medium was replaced with fresh medium every 48 h. After 2 d of differentiation, the cells were maintained in a medium containing 1 μg/mL insulin and 10% FBS, which was replaced every 2 d for 8 d. Cells from passages 5–7 were used for further experiments.

### 4.4. Cell Viability Assay

We performed the Cell Counting Kit-8 (CCK-8) assay to investigate the toxic effects of HPWE on 3T3-L1 cells. Briefly, the cells were seeded in a 96-well plate and cultured for 1 d with various concentrations of HPWE, and the cell viability was determined using the CCK-8 assay (Dojindo Molecular Technologies, Rockville, MD, USA). The culture medium was replaced with medium containing the CCK-8 solution (9:1) for 30 min, and the optical density was determined at 450 nm using a spectrophotometer (SpectraMax iD3; Molecular Devices, Sunnyvale, CA, USA).

### 4.5. Oil Red O Staining

Cells were cultured with different concentrations of HPWE for specific periods. Oil Red O staining (Sigma-Aldrich) was performed to observe lipid accumulation. Briefly, after the cells were incubated, they were rinsed twice with phosphate-buffered saline. The cells were fixed with 3.7% formaldehyde for 5 min, rinsed with distilled water, and stained with 0.5% Oil Red O in 60% isopropanol for 30 min. Images of the stained fat droplets in the adipocytes were captured using an inverted microscope (Leica Microsystems, Wetzlar, Germany). The dye was washed using 100% isopropanol, and the samples were transferred to a 96-well plate to measure the absorbance at 510 nm using a spectrophotometer (SpectraMax iD3; Molecular Devices, Sunnyvale, CA, USA).

### 4.6. RNA Isolation and Reverse Transcription-Quantitative Polymerase Chain Reaction (RT-qPCR)

RT-qPCR was conducted to measure the mRNA levels. TRIzol reagent (Invitrogen, Waltham, MA, USA) was used to isolate total RNA from the cells, according to the manufacturer’s recommendations. Briefly, after washing with PBS, the cells were lysed with TRIzol reagents in the recommended volume, and then chloroform was added. After treatment with chloroform, the cell lysates were incubated on ice for 10 min. Next, the cell lysates were centrifuged at 15,000 rpm at 4 °C for 10min. The supernatants were isolated in a new tube. After adding 2-propanol, the crude RNAs were spun down at 15,000 rpm at 4 °C for 10 min. Then, it was washed with 75% ethanol in DEPC water. The obtained pure RNA was quantified by NanoDrop™ 2000 (Thermo Scientific). We reverse-transcribed 1 μg total RNA using the RevertAid First Strand cDNA Synthesis Kit (Thermo Scientific), according to the manufacturer’s instructions. Primers were generated using online Primer3 software (version 2.6.1) [47] and are listed in Table 1. SYBR green-based RT-qPCR was conducted using a QuantStudio™ 5 real-time PCR System (Thermo Scientific) and PowerUp™ SYBR™ Green Master Mix (Thermo Scientific). All sample mixtures were run in triplicate, and the data were analysed using the 2^−ΔΔCT^ method [48]. β-actin was used as internal control.

### 4.7. Western Blotting

Total protein was isolated from 3T3-L1 cells using RIPA lysis buffer (Cell Signaling Technology, Danvers, MA, USA) containing a protease inhibitor. After a 10-min incubation period on ice, the proteins were extracted from the lysate by centrifugation at 15,000× *g* for 15 min. The protein concentration in the lysates was determined using a Detergent-Compatible (DC) Protein Assay Kit (Bio-Rad, Hercules, CA, USA). We loaded 30 µg total protein of each sample on a 7.5–15% sodium dodecyl sulphate-polyacrylamide gel for separation. The proteins were transferred to polyvinylidene fluoride membranes (Merck Millipore, Darmstadt, Germany). The membranes were incubated with appropriate primary and HRP-coupled secondary antibodies. The primary antibodies were diluted 1:1000 and HRP-conjugated secondary antibodies were diluted 1:3000 in 5% skim milk TBST. Clarity Western ECL Substrate (Bio-Rad) was used for development, and a ChemiDoc XRS+ system (Bio-Rad) was used for visualization.

### 4.8. Animal Experiments

This study was performed according to the recommendations of the Standard Protocol for Animal Studies of the Department of Laboratory Animal Resources, Yonsei Hospital Biomedical Research Institute. The experimental protocol was approved by the Institutional Animal Care and Use Committee (IACUC) of Yonsei Hospital Biomedical Research institute (Permit No. 2021-0183). All efforts were made to minimize suffering, stress/discomfort, and the number of animals. The maximum caging density was five mice from the same litter and sex starting from weaning. All materials, including lids, feeders, bottles, bedding, and water were autoclaved before use. Six-week-old male C57BL/6 mice were obtained from ORIENT BIO (Seongnam, Korea) and acclimatised for 1 week under the following conditions: 12/12-h light and dark cycle, controlled temperature (22–24 °C), and humidity 50–60%. The mice were provided with a laboratory diet and water ad libitum. Control mice were fed a normal diet (13.2% energy from fat; LabDiet 5053; LabDiet, St. Louis, MO, USA). To induce obesity, the mice were fed a high-fat diet (HFD; D12492; Research Diets, New Brunswick, NJ, USA), providing 60% energy from fat. The mice were randomly divided into the following five groups (n = 5 for each group): vehicle (standard diet + water), HFD (HFD + water), and 10, 50, and 100 mg per kg (mpk) HPWE (HFD + HPWE). The mice were weighed every 3 d. The treatments were administered 100 μL five times a week for 36 d via intragastric gavage using an oral zonde without anesthesia. No blinding was performed at any stage of this study.

### 4.9. ARRIVE Guidelines

A pre-clinical study was designed as a prospective, randomized, and blinded trial to assess the anti-obesity effects of HPWE. The sample size for each group was determined using G-power software (version 3.1.9.7) based on preliminary studies. It was concluded that three mice per group were sufficient to evaluate the effects of HPWE, with an additional two mice included to account for potential unexpected mortality. However, no mortality was observed during the experimental period. The allocation of animals to treatment groups was conducted using a computer-generated randomization tool (https://www.randomizer.org/, accessed on 1 December 2021). Each animal was assigned a unique identification number, and cages were numbered according to their position on the rack. The following parameters were evaluated: body weight, micro-CT, and tissue extraction.

### 4.10. Morphology of Adipose Tissue Samples

After feeding and drug administration, the mice were anaesthetized using alfaxan (Jurox Inc., Kansas City, MO, USA) and sacrificed by cervical dislocation. Epididymal WAT was separated and weighed using an electronic balance. Images of the epididymal WAT were captured using a digital camera with a 30-cm scale ruler.

### 4.11. Microcomputed Tomography (Micro-CT)

Abdominal fat volume was measured using a microCT system, and an in vivo micro-tomography system (in vivo Micro-CT, Skyscan 1276, SKYSCAN N.V., Kontich, Belgium) was used 1 d before the end of the experiment. We measured the grey color intensity of the tissues in the mouse abdomen using the CtAn program (Bruker-microCT Ct Analyzer, Kontich, Belgium) and based on the intensity (measured using the threshold method), each region was divided into lumbar vertebrae, lean tissue, adipose tissue, and skin. The extracted abdominal adipose tissue was reconstructed into a three-dimensional structure, and the structure was used for measuring the volume. The threshold was set to 20, and the image was processed by removing the bone and muscle tissues and acquiring an image of only the fat tissue.

### 4.12. Haematoxylin and Eosin Staining

The isolated epididymal WAT samples were fixed in 3.7% formaldehyde for at least 24 h, embedded in paraffin, and cut into 4-μm-thick sections using a sectioning machine (Leica Biosystems, Barrington, IL, USA). Next, hematoxylin and eosin staining were performed, images were captured using an Aperio AT2 slide scanner (Leica Biosystems, Barrington, IL, USA), and the size of adipocytes was measured using the ImageJ software (version 1.54k).

### 4.13. Liquid Chromatography-Mass Spectrometry (LC-MS/MS)

The saponarin was quantified using a Rapid LC-MS/MS spectrometer vanquish system (Thermo Fisher Scientific) in conjunction with a TSQ Altis Plus triple quadrupole mass spectrometer (Thermo Fisher Scientific) operating in multiple reaction monitoring (MRM) mode. A 1 μL sample was introduced at a flow rate of 0.2 mL/min and separated using a Cortects C18 column (2.1 × 50 mm, 1.6 μm, Waters Co., Milford, MA, USA) with the column temperature maintained at 45 °C. The mobile phases consisted of 0.1% Formic acid in distilled water (Phase A) and 0.1% Formic acid in acetonitrile (Phase B), with a flow rate of 0.2 mL/min. Quantitative analysis was conducted using mass spectrometry (MS/MS). The mass spectrometer operated with heated electrospray ionization (H-ESI) in negative mode, and the saponarin precursor ion had an *m*/*z* value of 593.175. Calibration curves for saponarin were established within the range of 0 to 500 ng/mL, achieving an R^2^ of 1. The concentration of saponarin in the HPWE was determined at the National Instrumentation Center for Environmental Management (NICEM) at Seoul National University.

### 4.14. Statistical Analysis

All data are presented as mean ± standard deviation. Each experiment included three replicates for each variable and was performed 3–5 times. Figure 1, Figure 2, Figure 3, Figure 4 and Figure 5 present the results of representative experiments. Statistical significance was determined using Student’s *t*-test and one-way analysis of variance (ANOVA), followed by Tukey’s honest post hoc test using the professional Statistical Package software GraphPad Prism 5.0 (GraphPad Software, San Diego, CA, USA). significance was set at *p* < 0.05.

## 5. Conclusions

To the best of our knowledge, this study is the first to report the anti-obesity effects of HPWE. In vitro, HPWE reduced lipid accumulation in 3T3-L1 cells. Moreover, HPWE treatment suppressed the expression of transcription factors involved in adipogenesis and decreased the levels of adipogenesis-associated molecular markers at both the mRNA and protein levels. In vivo, HPWE administration regulated body weight in mice by reducing the mass of epididymal white adipose tissue (WAT), abdominal fat deposits, and the size of adipocytes within epididymal WAT. These effects are likely attributable to the high concentration of saponarin in HPWE. However, the limitations of our study are that we have not conducted clinical trials on humans and have not identified bioactive compounds other than saponarins. Nonetheless, these findings suggest that HPWE has potential as a preventive agent against obesity in both in vitro and in vivo models.

## Figures and Tables

**Table 1:** Primer sequences used in this study.

Target Gene	Forward Primer (5′–3′)	Reverse Primer (5′–3′)
*C/EBPα*	GAACAGCAACGAGTACCGGGT	GCCATGGCCTTGACCAAGGAG
*PPARγ*	GCCTTTTGGTGACTTTATGGA	GTAGCAGGTTGTCTTGAATG
*FABP4*	GGATGGAAAGTCGACCACAA	TGGAAGTCACGCCTTTCATA
*FAAH*	ACTTGGACGTGGTGCTAACC	GCCTATACCCTTTTTCATGCCC
*Plin 1*	GCGGAATTTGCTGCCAACACTC	AGACTTCTGGGCTTGCTGGTGT
*β-Actin*	AGGCTGTGCTGTCCCTGTAT	ACCCAAGAAGGAAGGCTGGA

## Data Availability

The data presented in this study are available upon request from the corresponding author.

## Supplementary Materials

The following supporting information can be downloaded at: https://www.mdpi.com/article/10.3390/ijms26209870/s1.
