# Supplementary material for: Hibiscus syriacus Bud ‘Pyeonghwa’ Water Extract Inhibits Adipocyte Differentiation and Mitigates High-Fat-Diet-Induced Obesity In Vivo
Source: Int J Mol Sci. 2025 Oct 10;26(20):9870. doi: 10.3390/ijms26209870 (PMC12562410; doi:10.3390/ijms26209870)
Supplement: Supplementary file 1 [file ijms-26-09870-s001.zip › ijms-3818861-supplementary.pdf]

International Journal of
Molecular Sciences

MDPI

Supplementary Materials

Hibiscus syriacus Bud 'Pyeonghwa' Water Extract Inhibits Adipocyte Differentiation and Mitigates High-Fat-Diet-Induced Obesity In Vivo

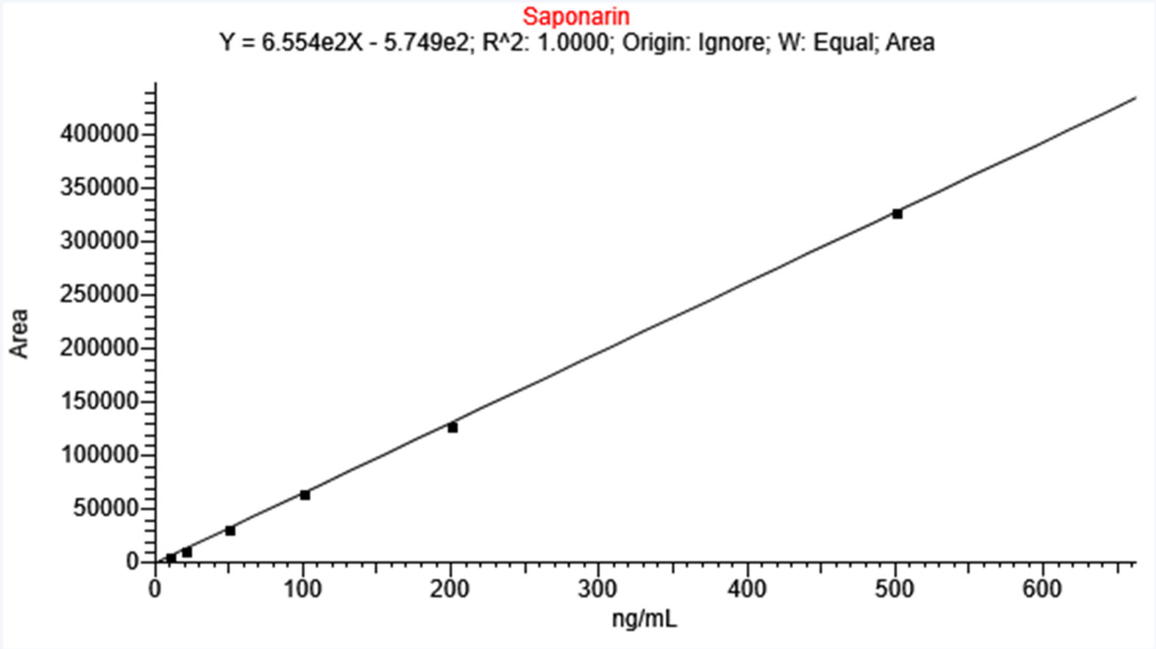

Supplementary Figure S1. The saponarin compound calibration for Quantitative Analysis using LC-MS/MS.

 https://doi.org/10.3390/ijms26209870
